# Symmetric vs. Asymmetric Stem Cell Divisions: An Adaptation against Cancer?

**DOI:** 10.1371/journal.pone.0076195

**Published:** 2013-10-29

**Authors:** Leili Shahriyari, Natalia L. Komarova

**Affiliations:** Department of Mathematics, University of California Irvine, Irvine, California, United States of America; Indian Institute of Toxicology Reserach, India

## Abstract

Traditionally, it has been held that a central characteristic of stem cells is their ability to divide asymmetrically. Recent advances in inducible genetic labeling provided ample evidence that symmetric stem cell divisions play an important role in adult mammalian homeostasis. It is well understood that the two types of cell divisions differ in terms of the stem cells' flexibility to expand when needed. On the contrary, the implications of symmetric and asymmetric divisions for mutation accumulation are still poorly understood. In this paper we study a stochastic model of a renewing tissue, and address the optimization problem of tissue architecture in the context of mutant production. Specifically, we study the process of tumor suppressor gene inactivation which usually takes place as a consequence of two “hits”, and which is one of the most common patterns in carcinogenesis. We compare and contrast symmetric and asymmetric (and mixed) stem cell divisions, and focus on the rate at which double-hit mutants are generated. It turns out that symmetrically-dividing cells generate such mutants at a rate which is significantly lower than that of asymmetrically-dividing cells. This result holds whether single-hit (intermediate) mutants are disadvantageous, neutral, or advantageous. It is also independent on whether the carcinogenic double-hit mutants are produced only among the stem cells or also among more specialized cells. We argue that symmetric stem cell divisions in mammals could be an adaptation which helps delay the onset of cancers. We further investigate the question of the optimal fraction of stem cells in the tissue, and quantify the contribution of non-stem cells in mutant production. Our work provides a hypothesis to explain the observation that in mammalian cells, symmetric patterns of stem cell division seem to be very common.

## Introduction

The ability of stem cells to divide asymmetrically to produce one stem and one non-stem daughter cell is often considered to be one of the defining characteristics of stemness. On the other hand, there is ample evidence suggesting that adult stem cell can and do divide symmetrically [1], [2].

**Figure 1:**
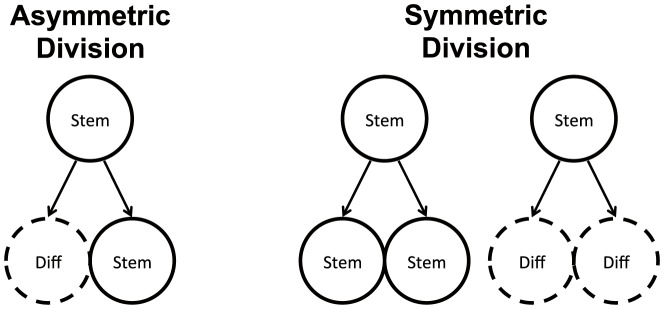
Symmetric and asymmetric stem cell divisions. In the asymmetric division model, a stem cell produces one differentiated cell and one stem cell. In the symmetric division model, a stem cell produces two differentiated cells or two stem cells.

Two basic models of stem cell divisions are discussed in the literature, see Figure 1. The asymmetric model suggests that the homeostatic control of the stem cell pool is maintained at the level of single cells, whereby each stem cell produces a copy of itself plus one differentiated cell [4]–[6]. The mechanisms involved in asymmeric divisions have been characterized in some detail in Drosophila, and involve regulation of cell polarity and orientation with respect to the stem cell niche [3]. From the engineering prospective, this model has the advantage of keeping the stem cell population level steady. An obvious disadvantage is its inability to replenish the stem cell pool in case of injury. This problem is naturally solved by the symmetric model, which maintains homeostatic control at the population level, rather than at the individual cell level. There, stem cells are capable of two types of symmetric divisions: a proliferation division resulting in the creation of two stem cells, and a differentiation division resulting in the creation of two differentiated cells [7]–[10]. Differentiation/proliferation decisions are though to be under control of numerous signals emanating from the surrounding tissue and the stem cells themselves [11]–[17], [19]–[29]. Stem cell cycle regulation is thought to play a key role in the orchestrating of stem cell renewal [18].

Uncovering division patterns of stem cells has been subject of intense research in the last fifteen years. Some of the first quantification of the division strategies *in vitro* comes from the work of Yatabe *et al* who tracked methylation patterns in the dividing cells of the colon crypts [30]. The analysis of the complex methylation patterns revealed that crypts contain multiple stem cells that go through “bottlenecks” during the life of the organism, which suggests that symmetric divisions are part of the picture. Another piece of evidence comes from experiments with chimeric mice to determine the dynamics of polyclonality of crypts. Initially polyclonal crypts eventually become monoclonal, which suggests that symmetric divisions must occur [31], [32]. By means of radiotherapy-induced mutations, it was found that a significant fraction of the somatic mutations in human colon stem cells are lost within one year [33].

An important advance in quantification of symmetric vs antisymmetric divisions became possible with the invention of inducible genetic labeling [34]. This technique provides access to lineage-tracing measurements, from which the fate of labeled cells and their clones can be tracked over time. By means of the quantitative analysis of long-term lineage-tracing data [10], [35], it has been shown that the rate of stem cell replacement is comparable to the cell division rate, implying that symmetric cell divisions contribute significantly to stem cell homeostasis [36], [37]. Ref. [38] provides a review of the recent evidence of symmetric divisions in mammalian intestinal stem cells, spermatogenesis and epithelial tissues such as hair follicles [35], [39].

These new findings reveal that contrary to the previous thinking, adult tissue stem cells are often lost (e.g. by differentiation) and replaced in a stochastic manner. This notion challenges the traditional concept of the stem cell as an immortal, slow-cycling, asymmetrically dividing cell [34].

In paper [38], an important question is raised: Why should mechanisms of tissue maintenance so often lean toward symmetric self-renewal? One answer comes from recognizing the ability of symmetrically-dividing stem cells to respond to injury thus ensuring a robust mechanism of tissue homeostasis. It however could be argued that the symmetric divisions are “switched on” in response to a sudden stem cell loss, and the asymmetric division strategy is employed in the course of normal homeostasis.

In the present paper, we explore an alternative hypothesis, which gives an additional “reason” for the tissue architecture favoring symmetric divisions. As a starting point, we note that in both symmetric and asymmetric division types, a dysregulation may lead to the loss of homeostatic control and an unchecked growth of cells. A disruption in the control of proliferation/differentiation decisions can tip the balance and lead to abnormal stem cell expansion [40]. It has also been shown that disruption of asymmetric divisions can be responsible for cancerous growth of undifferentiated cells [41]–[45].

Here, we examine the symmetric and asymmetric divisions in the context of producing mutations. Many cancerous transformations start off by an inactivation of a tumor-suppressor gene [46]. This is the famous two-hit process discovered by Knudson [47], [48] and studied by many laboratories as well as theoretically. We ask the following question: from the point of view of two-hit mutant generation, what type of stem cell divisions is advantageous for the organism? What frequency of symmetric vs asymmetric divisions can maximally delay the stochastic generation of a dangerous mutant? To this end, we consider a continuous range of strategies with mixed type divisions and explore how the frequency of symmetric vs asymmetric divisions affects the generation of mutations.

In this paper, we use both numerical simulations and analytical methods to study symmetric and asymmetric stem cell divisions in the context of mutation production. Other theoreticians have explored stem cell dynamics by means of deterministic stem cell modeling and stochastic numerical simulations [49]–[66]. A great review of many modeling approaches is provided in Ref. [67]. Ref. [68] studied the dynamics of mutation spread in development and showed that susceptibility to late-life cancers may be influenced by somatic mutations that occur during early development. Ref. [69] considered a model of stem cell dynamics, and calculated the rates of stochastic elimination (or washing out) of mutants. In this model, stem cells can proliferate symmetrically and differentiation is decoupled from proliferation. Ref. [70] considered the question of mutation generation by stem cells and found that mutations that increase the probability of asymmetric replication can lead to rapid expansion of mutant stem cells in the absence of a selective fitness advantage. In Ref. [71] it is shown that symmetric stem cell divisions can reduce the rate of replicative aging.

In the present paper, we concentrate on the optimization problem of tissue architecture in the context of delaying double-hit mutant production, and focus specifically on symmetric and asymmetric stem cell divisions. We consider a stochastic model of double-hit mutant generation, and ask several questions related to evolutionary dynamics of mutations. What type of divisions is optimal? What cell types contribute the most to double-hit mutant generation? What is the optimal fraction of stem cells that delays carcinogenesis?

## Results

### Set-up

We consider a two-compartment, agent-based model of stem cells and transit-amplifying (TA) cells. The stem cells are capable of both symmetric and asymmetric divisions (see Figure 1). The relative proportion of symmetric divisions can vary and is denoted by the symbol 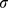 (see Table 1), where 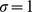 means that all divisions are symmetrical, and 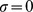 means that stem cells only divide asymmetrically. The symmetric divisions can be of two types, proliferation and differentiation. The type of symmetric division is defined by a regulatory mechanism which assures an approximately constant level of stem cells (see Methods). The total population (which includes both stem cells, 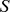, and TA cells, 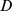) is denoted by 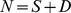. An important parameter is 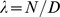, which defines the proportion of stem cells with respect to TA cells: 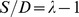.

**Table 1:** Model parameters.

Notation	Description
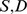	Number of stem and TA cells
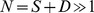	Total population size
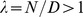	Inverse relative number of TA cells
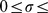 1	Proportion of symmetric stem cell divisions
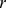 ≲ 1, 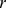 ≳ 1	Fitness of one-hit mutants
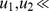 1	Mutation rates leading to the acquisition of first and second hits
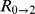	Tunneling rate
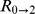	For immortal DNA strand hypothesis: Prob. of a mutation in the stem offspring
	rather than the TA offspring of a stem cell upon an asymmetric division

Notations used in the text and their brief description.

We assume that the non-stem cells can die, and that all cell types have a chance to divide. Each time a division happens, there is a probability, 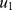, that one of the daughter cells is a one-hit mutant. The first mutation can alter the properties of the cell. We assume that the relative fitness of one-hit mutants is given by parameter 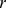 (while the fitness of all wild-type cells is given by 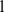). The fitness parameter defines the relative probability of the given cell-type to be chosen for division. In this paper we consider a range of fitness values, 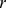, such that the one-hit mutants can be disadvantageous compared to wild-type cells (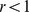), neutral (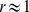), or even slightly advantageous (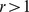). When a one-hit mutant divides, it has the probability 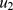 to give rise to a two-hit mutant. Two-hit mutants are transformed cells which have a potential to give rise to a cancerous tissue transformation.

The generation of two-hit mutants is normally considered to be a rate-limiting step in cancer initiation. Once such a mutant is produced, it may break down homeostatic control and result in a wave of clonal expansion, followed by further transformations. It is this first step, the creation of a double-hit mutant, that we focus on in this paper. We investigate how the timing of such a mutant production depends on the tissue architecture, and specifically, on the symmetry of stem cell divisions.

In order to gain analytical insights, a slightly simplified stochastic process was considered (see the Methods Section) which gave predictions that are in excellent agreement with the computational model.

### Tunneling rates

**Figure 2:**
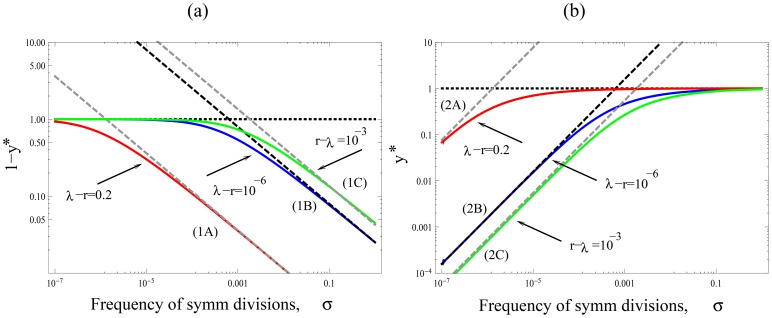
The six different approximation regimes (Table 2) for solutions of system (2–3). Plotted is the quantity (a) 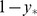 and (b) 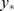 as a function of the frequency of symmetric divisions, 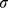, for three different values of 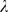 (solid lines), together with the approximations given by the formulas in Table 2. Approximations 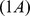, 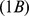, and 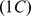 are best demonstrated in panel (a), where the quantity 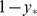 is plotted. Approximations 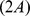, 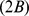, and 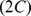 are best demonstrated in panel (b), where the quantity 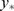 is plotted. The other parameters are 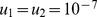, 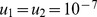.

While the detailed temporal dynamics of double-mutant production is given in the Methods Section, here we present the results for the so-called “tunneling rates” - the rates at which the stem cell system of a given size produces double-hit mutants (assuming that one-hit mutants drift at relatively low levels). Denoting the tunneling rate as 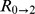 (where the subscript suggests that the system transfers from all wild-type, “zero-hit”, state to a system containing two-hit mutants), we have
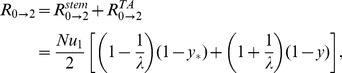
(1)where quantities 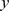 and 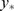 satisfy the system
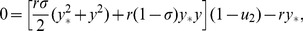
(2)

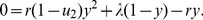
(3)The time to produce double-hit mutants is distributed exponentially with the mean
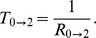
Formula (1) describes the generation of double-hit mutants in the stem cells (the first term on the right) and in TA cells (the second term of the right). Several limiting cases are presented in Table 2 and illustrated in Figure 2.

**Table 2:** Important limiting cases for the tunneling rate (formula (1)).

Regime	Description	Conditions	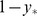	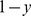
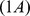	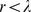 , symm+asymm	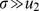 , 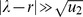 , 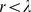	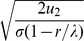	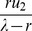
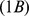	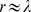 , symm+asymm	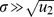 , 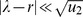	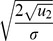	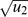
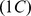	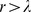 , symm+asymm	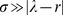 , 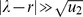 , 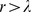	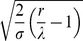	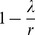
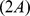	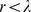 , asymm	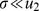 , 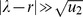 , 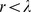	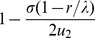	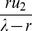
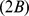	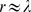 , asymm	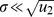 , 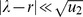	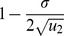	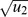
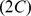	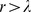 , asymm	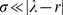 , 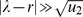 , 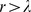	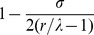	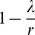

The notations for the six different regimes refer to Figure 2.

Predictions of formula (1), as well as the more precise equation (11), have been compared with stochastic numerical simulations, and found to be in excellent agreement with them, see below.

### Double-hit mutants are produced slower under symmetric compared to asymmetric divisions

**Figure 3:**
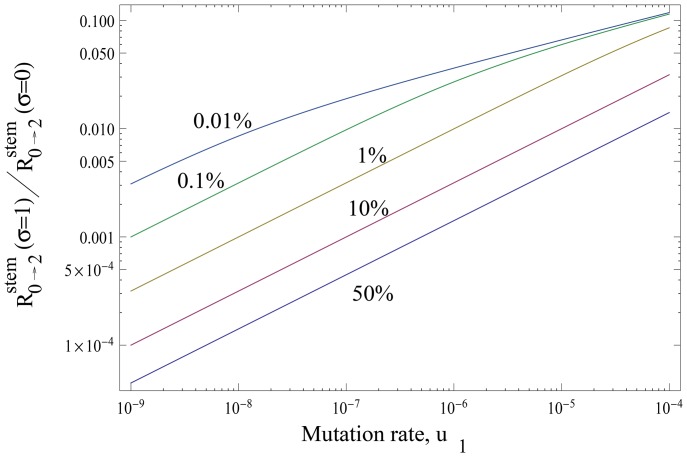
The reduction in the rate of double mutant production in stem cells with symmetric divisions compared to stem cells with asymmetric divisions only. Plotted is the quantity in formula (4) as a function of the mutation rate, 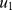. The percentage of the stem cells in the whole population (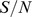) is marked next to the lines. The other parameters are 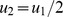, 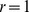.

An important question is how the fraction of symmetric divisions (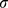) affects the rate of double-mutant production. We can see that the production of double-mutants by non-stem cells does not depend on 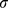, the frequency of symmetric divisions. On the other hand, the production by stem cells is crucially affected by this parameter. Our formulas show clearly that the rate of tunneling grows as 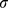 decreases, and it is the highest when 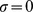, the case of purely asymmetric divisions. This means that in order to minimize the rate of double-hit mutant formation, one needs to maximize the share of symmetric divisions. In Figure 3 we plot the quantity
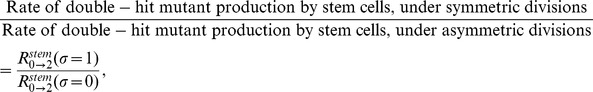
(4)for different percentages of stem cells. We can see that for realistic ranges of the mutation rates, the difference is at least 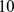-fold, and can be as high as 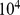-fold, with the symmetrically dividing stem cells producing double-hit mutants slower than asymmetrically dividing cells.

**Figure 4:**
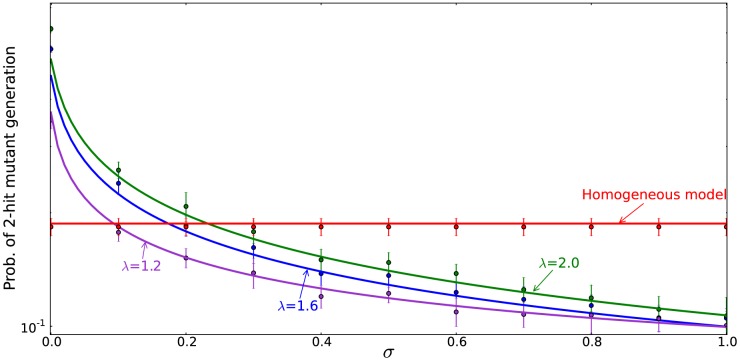
The probability of double-hit mutant generation as a function of 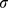, the probability of symmetric stem cell divisions. The results of numerical simulations are presented as points connected with dotted lines (standard deviations are included). Analytical results are given by solid lines (formula (11). The horizontal line represents the calculations for the homogeneous model. We ran 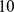 batches of 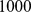 runs. The parameters are 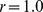, 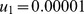, 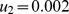, 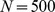.

Figure 4 compares the analytical findings for the double-hit mutant production dynamics with the numerical simulations. We ran the stochastic numerical model (see Methods) for a fixed number of time-steps, and recorded whether or not a double-hit mutant has been generated. Repeated implementation of this procedure produced a numerical approximation of the probability of double-hit mutant generation, which is plotted (together with the standard deviations) as a function of 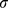, the probability of symmetric divisions, for three different values of 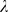, which measures the fraction of stem cells. Clearly, the probability of mutant generation in the course of a given time-interval is a decaying function of 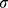.

Another result that follows from our computations is the comparison of the double-mutant production in a hierarchical (stem cells plus TA cells) model compared with the conventional, homogeneous model that has been extensively studied [72]–[75]. It turns out the hierarchical model with purely asymmetric divisions always produces mutants faster than the homogeneous model. For the hierarchical model with purely symmetric divisions the result depends on the fitness of one-hit mutants. For disadvantageous one-hit mutants whose fitness satisfies 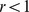, 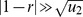, the hierarchical model with purely symmetric divisions produces double-mutants faster, and for neutral and advantageous mutants, it produces double-hit mutants slower than the homogeneous model. In figure 4 we can see that for 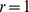 (neutral one-hit mutants), hierarchical models with a sufficiently large values of 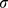 are characterized by slower double-hit mutant generation compared to the homogeneous model (the horizontal line).

**Figure 5:**
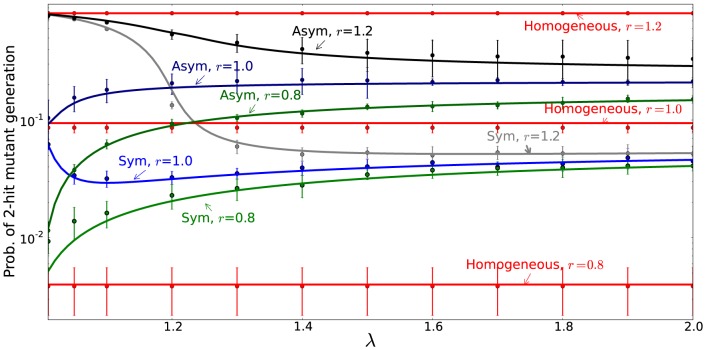
The probability of double-mutant generation as a function of 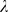, the ratio of TA cells to the total number of cells. As in Figure 4, the results of numerical simulations are presented as points connected with dotted lines (standard deviations are included), and the analytical results are given by solid lines (formula (11)). The horizontal lines represent the calculations for the homogeneous model. We ran 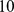 batches of 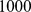 runs. Plotted is the probability of double-mutant generation as a function of 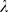, for purely symmetric (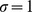) and purely asymmetric (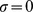) models, for three different values of 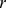. The parameters are 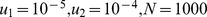.

Figure 5 shows additional results of simulations (together with our analytical calculations), where for three different values of 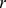 (one-hit mutant fitness) the probability of double-hit mutant generation is plotted as a function of 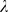. The values 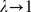 corresponds to a vanishingly low fraction of stem cells in the system, while 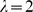 corresponds to 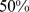 of all cells being stem cells. We show purely symmetric (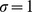) and purely asymmetric (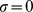) cases. For fixed mutation rates and populations sizes, the homogeneous model is characterized by only one parameter, 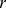, which is the fitness of one-hit mutants. The probability of double-hit mutant generation strongly depends on whether these intermediate mutants are disadvantageous (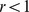), neutral (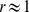), or advantageous (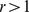). In contrast to the homogeneous model, the hierarchical model contains two additional parameters, 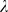 (the ratio of TA cells and the total population) and 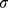 (the probability of symmetric divisions). We can see that these two parameters affect the probability of double-hit mutant generation at least as strongly as the fitness 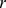 does. The influence of 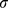 is clear: the more the fraction of symmetric divisions, the slower double-hit mutants are produced. Next, we examine the role of the stem cell to TA cell ratio.

### The optimal fraction of stem cells

Let us consider an optimization problem for the tissue design, with the goal to delay the production of double-hit mutants. What is the optimal fraction of stem cells that the population should maintain? Analysis of the tunneling rates for a hierarchical model with purely symmetric divisions suggests that the optimal fraction of stem cells depends on the fitness of the one-hit mutants. If the one-hit mutants are disadvantageous (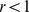, 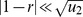), then the tunneling rate grows with the parameter 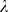. In other words, in order to minimize the rate of double-mutant production, one would need to keep the stem cell pool as small as possible.

For neutral and advantageous intermediate mutants, where the symmetric division model gives rise to the lowest double-mutant production rate compared to the homogeneous model and the hierarchical model with asymmetric divisions, this rate is minimized for a particular fraction of stem cells. This fraction is defined by the mutation rate 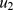 in the neutral case, and by the fitness of the intermediate mutants in the case of weakly advantageous mutants. For neutral one-hit mutants (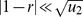), the optimal value of 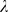 is given by
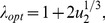
(5)and for weakly advantageous mutants with 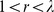, 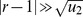, we have
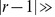
(6)For example, for the biologically most relevant case of neutral one-hit mutants, the optimal fraction of stem cells is approximately 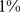 of the total population, assuming 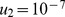.

**Figure 6:**
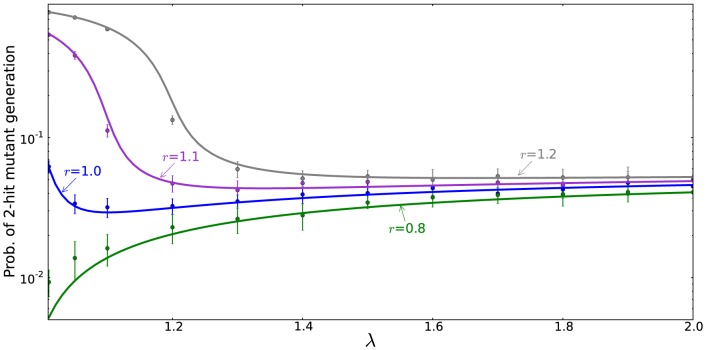
The probability of double-hit mutant generation in the symmetric division model. The case of symmetrically dividing stem cells, same as in Figure 5.

These results are illustrated in Figure 6. In this plot, we can see for 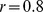 the probability of having a doubly mutated cell (after a given time-span) is an increasing function of 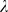, as predicted. For the case of 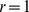, the numerical simulation in Figure 6 shows that 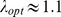 (compared with 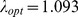 predicted by formula (5)). For the case 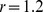, formula (6) gives 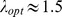, which approximately coincides with the numerical optimum. In the case of advantageous mutants however the minima of 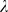 are very shallow.

### Do mutations in TA cells produce double-mutants?

**Figure 7:**
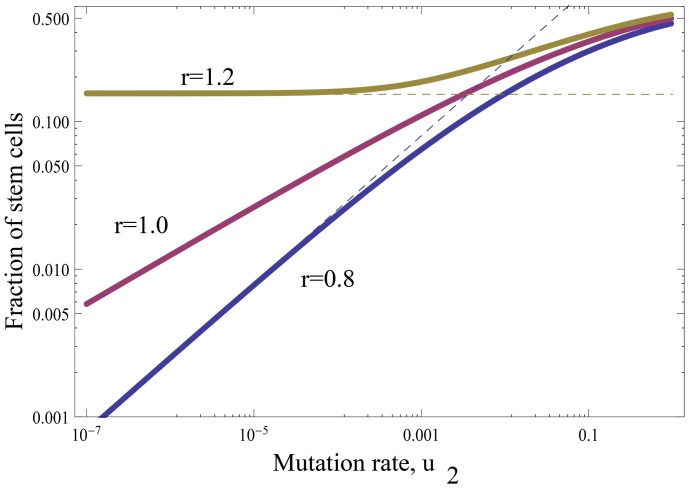
The threshold fraction of stem cells corresponding to stem and TA cells contributing equally to double-hit mutant production. The quantity 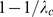, which corresponds to 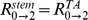, is plotted as a function of the mutation rate, 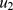, for three different values of 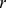, and 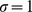. For the fraction of stem cells above these values, stem cells have a higher contribution to the rate of double-mutant production compared to the non-stem cells. Thin dashed lines show the approximations of equation (8).

Let us compare the relative contributions to the double-mutant production rate coming from stem cells and TA cells, equation (1):
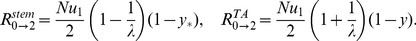
(7)The contribution from the TA cells grows as the fraction of TA cells increases. In Figure 7 we plot the fraction of stem cells (given by 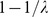) that corresponds to 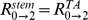. We can see that for the mutation rates around 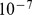, this fraction is about 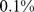 for disadvantageous intermediate mutants, about 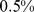 for neutral mutants, and about 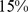 for advantageous mutants. This means that as long as the fraction of stem cells in the population is lower than these threshold values, TA cells contribute *more* to the production of double-hit mutants than stem cells. This threshold fraction grows for larger mutation rates, making it easier for TA cells to contribute significantly to the double-hit mutant production. An analytical approximation for the threshold value of 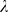 can be found for small values of mutation rates, such as
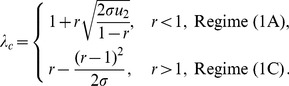
(8)


Next we address the question of optimization assuming that only mutations acquired by stem cells are dangerous and can lead to further malignant transformations. In this case, the rate of mutant production is given by 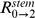, equation (7). It is easy to show that this quantity is maximized by asymmetric divisions only (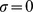), and it is minimized by symmetric divisions of stem cells (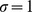). Thus the message of this paper does not change if only stem cell mutations are assumed to contribute to carcinogenesis.

### The immortal DNA strand hypothesis: an additional mutation-reducing mechanism

The immortal DNA strand hypothesis was proposed in by John Cairns in 1975 as a mechanism for adult stem cells to minimize mutations in their genomes [76]. It is proposed that upon asymmetric division, the DNA of a stem cell does not segregate randomly, but instead the daughter stem cell retains a distinct template set of DNA strands (called the parental strand). As a result, stem cells pass mutations arising from errors in DNA replication on to their TA daughters, which soon terminally differentiate. Support for the immortal DNA strand hypothesis has been reported by several groups, see e. g. [77], [78], while other authors maintain that it does not yet have a convincing experimental confirmation [79].

**Figure 8:**
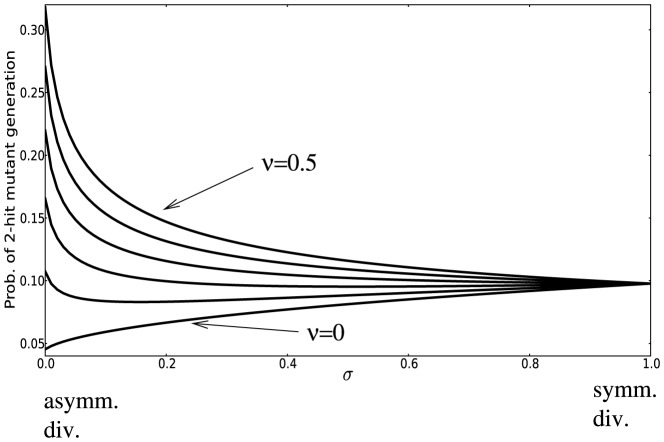
The immortal DNA strand hypothesis. The probability of double-hit mutant generation is calculated for a particular set of parameters as a function of 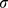 (the probability of symmetric divisions), according to formula (21. For 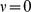 the minimum corresponds to at 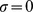 (asymmetric divisions only), for 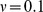 and 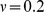 we have an intermediate minimum at 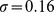 and 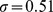 respectively, and for higher values of 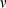 the minimum is reached for 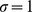 (symmetric divisions). Here, 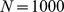, 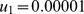, 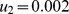, 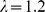, 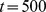, and the parameter 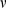 varies from 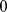 to 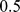 in increments of 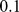.

It is possible to incorporate this mechanism into our model. We introduce a parameter 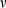, which quantifies the probability of a mutation occurring in the TA offspring of an asymmetrically dividing stem cell rather than in its TA offspring. The case 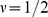 corresponds to a complete symmetry between stem and TA offspring, and 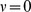 to the situation where the parental strand can never acquire mutations. In Figure 8 we plot the probability of double-hit mutant generation as a function of 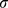, the probability of symmetric divisions, for different values of 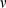 (see formula (21)). It is hardly surprising that for 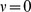, the minimum corresponds to at 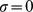, asymmetric divisions only. In this case, asymmetrically dividing stems do not accumulate mutations, while symmetrically dividing stems have a chance to acquire mutations. Because of this additional mechanism protecting against mutations, asymmetric divisions are the optimal strategy from the point of view of minimizing double-hit mutant accumulation. On the other hand, if 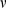 is relatively high, this mechanism is not sufficient to outweigh the inherently slower accumulation of mutants by symmetrically dividing cells, resulting in the optimal strategy with 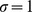. Intermediate values of 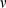 correspond to intermediate values of 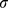, such that a mixture of symmetric and asymmetric divisions comprises the optimal strategy.

## Discussion

In this paper we found that symmetrically dividing stem cells are characterized by a significantly lower rate of two-hit mutant generation, compared to asymmetrically-dividing cells. This is especially important in the context of tumor-suppressor gene inactivation, which is one of the more common patterns of carcinogenesis. This provides an evolutionary framework for reasoning about stem cell division patterns.

In the literature, both types of stem cell divisions have been reported in various tissues. It has also been reported that the same stem cells are capable of both symmetric and asymmetric divisions. Whether a cell divides symmetrically or asymmetrically depends on factors such as the polarized organization of the dividing cell as well as the cell cycle length [80]. In Drosophila germ stem cells, cell division is asymmetric or symmetric depending on whether the orientation of the mitotic spindle is perpendicular or parallel to the interface between the stem cell and its niche [81]. Similarly, mammalian stem cells have been reported to employ both symmetric and asymmetric divisions to regulate their numbers and tissue homeostasis [82], [83]. A switch from a symmetric mode of divisions to the asymmetric model has also been reported to take place in development (see Refs. [84], [85] in the context of Drosophila).

The fact that the rate of double-hit mutant production is the lowest for symmetrically dividing cells does not in itself explain or predict any aspects of the tissue architecture. It however provides an alternative hypothesis for the observation that in mammalian tissues, symmetric patterns of stem cell division seem to be very common. The force of selection that comes from the cancer-delaying effect of such an architecture can be thought to have helped shape the observed division patterns. On the other hand, in more primitive organisms such as Drosophila, asymmetric stem cell divisions seem to dominate adult homeostasis (following the predominantly symmetric division patterns of development). Since cancer delay does not provide an important selection mechanism in the context of Drosophila, we can argue that this could help explain the observed differences.

### Symmetric divisions can have a cancer-delaying effect

The mathematical result obtained here is that symmetrically dividing cells appear to delay double-hit mutant production compared to an equivalent system with asymmetrically dividing stem cells. What is the intuition behind this finding? Double-mutants are generated by means of mutations that happen in singly-mutated cells. To understand this process, let us focus on the dynamics of single mutants. In particular, we concentrate on singly-mutated stem cells, because the fates of single mutations in TA cells are identical in the two models. What happens to a singly-mutated stem cell under the different division patterns?

**Figure 9:**
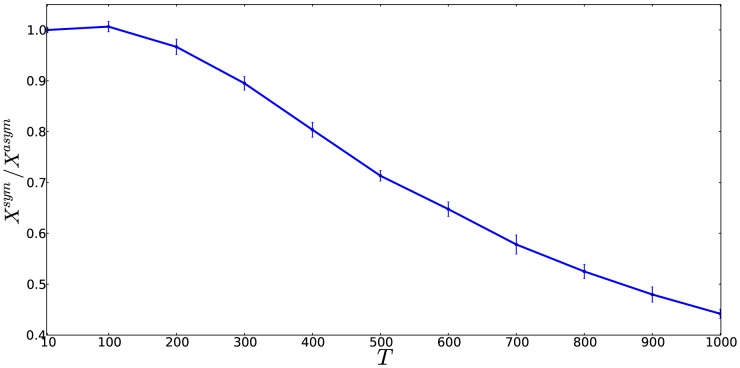
Why are symmetrically dividing stem cells produce mutants slower? The weight of a typical symmetrically dividing mutant stem cell lineage, 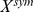, relative to the weight of an asymmetrically dividing mutant stem cell lineage, 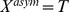, is plotted as a function of the number of stem cell divisions, 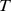. Here, 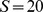, 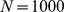, and 20 batches of 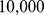 simulations were performed to calculate the mean and the standard deviation.

As noted by [86], if stem cells divide asymmetrically, then a mutation acquired in a stem cell will remain in the system indefinitely, because at every cell division, a new copy of the mutant stem cell will be generated. On the other hand, a mutant stem cell generated under the symmetric division model has a very different and much less certain fate. Each division of a mutant stem cell can result either in (1) elimination of the mutation from the stem cell compartment as a result of a differentiation, or (2) creation of an additional mutant stem cell as a result of a proliferation event, see also [86]. Superficially, it might look like the two processes might balance each other out. This intuition is however misleading. A lineage of mutant stem cells starting from a single mutant stem cell is much more likely to die out than to persist and expand. In fact, only 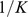 of all such lineages will expand to size 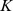. Half of the lineages will differentiate out after the very first division. Statistically there will be occasional, rare long-lived lineages, but the vast majority will leave the stem cell compartment after a small number of divisions. The production of those “lucky” long-lived mutants is not enough to counter-balance the great majority of the dead-end lineages that quickly exit the stem cell compartment. This is illustrated in Figure 9, which plots the “weight” (the net size of a lineage over time, 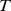) of a typical symmetrically dividing mutant stem cell, 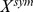, divided by the weight of a typical asymmetrically dividing mutant stem cell, 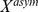. The latter quantity is simply given by 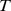, and the former quantity is a stochastic variable. We can see that the weight of symmetrically dividing mutant lineages is always lower than that of asymmetrically dividing lineages, which means that the former will have a lower probability to produce double-mutants offspring. We conclude that the uncertainty of fate of single mutant stem cells is the reason for the statistically longer time it takes for the symmetrically dividing stem cell model to produce a double-hit mutant.

Interestingly, the above argument can be made in a similar manner for disadvantageous, neutral, or advantageous mutants. In any of those cases, an asymmetrically dividing mutant stem cell remains in the population indefinitely. In the model with symmetric divisions, whenever a mutant stem cell is chosen for division, its probability to proliferate is similar to its probability to differentiate (in order to keep the homeostasis), and this the dynamics of each lineage is independent of its fitness (except that the frequency of updates is determined by the fitness of mutants; this is why the fitness parameter 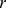 factors out of equations (2) and (12)).

We note that the effect of double-hit mutant production delay caused by symmetric divisions compared to asymmetric divisions is very significant. The difference in the tunneling rate which characterizes the time-scale of the process can be as high as 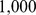-fold for tissues with 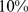 of stem cells and the mutation rate of 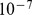 per gene per cell division.

### Can TA cells create double-hit mutants?

The model studied in this paper tracks single- and double-hit mutant production in both stem and TA cells. It is interesting to compare which mechanism (through stem cell single mutants or TA cell single mutants) contributes more to the double-mutant production? It turns out that as long as the fraction of stem cells is smaller than a threshold (or equivalently, if the fraction of the TA cells is larger than a threshold), non-stem cells contribute equally or more to the production of double-mutants. This threshold fraction depends on (1) the mutation rate and (2) the fitness of intermediate, one-hit mutants. For example, if the intermediate mutants are neutral and the mutation rate is 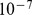 per gene per cell-division, then the threshold fraction of stem cells is about 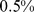 of the total population. In other words, mutations originating in non-stem cells are significant if stem cells comprise less than 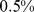 of the total population. This number is much higher if the intermediate mutants are advantageous, or if the mutation rate responsible for the second hit is higher. For 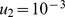, non-stem cells are the driving force behind double-mutant production as long as stem cells comprise less than about 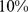 of the total population. This scenario is realistic in the presence of genetic instability, where inactivation of a tumor suppressor gene is likely to occur through a small-scale mutation of the first copy of the gene followed by a loss of heterozygocity event inactivating the second copy. The latter can happen a rate as high as 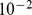 [87].

The arguments presented above clarify some aspects of the long-standing debate about the origins of cancer, see also Ref. [88]. It is sometime argued that TA cells are unimportant for cancer initiation, for the following (quantitative) reason, unrelated to biological evidence. Intuitively, it seems that double-hit mutants cannot be created among TA cells, because all one-hit mutants in the TA compartment will be washed away before they have a chance to acquire the second hit. As John Cairns writes, “…there are 256 exponentially multiplying cells that divide twice a day and are being replenished continually by the divisions of a single stem cell, none of these 256 cells will ever be separated from the stem cell by more than eight divisions, and the replication errors made in those eight divisions are destined, of course, to be discarded” [89]. The computations in this paper demonstrate that under some realistic parameter regimes, double-hit mutants can be created in the TA compartment, and TA cells statistically can contribute equally or more to double-hit mutant production compared to stem cells. The simple reason for this is as follows. Even though TA cells are short-lived, and getting a second mutation in a singly-mutated TA cell is unlikely, there are many more TA cells than stem cells. The low chance of double-mutant generation in a single TA cells can be outweighed by the fact that TA cells are a large majority, and single probabilities add up to create a significant effect.

### Cancer stem cell hypothesis

The question discussed above is purely mathematical, and deals with the simple possibility to acquire two hits in the TA compartment. A related biological question is whether mutations occurring in the TA compartment can lead to further carcinogenic transformations, which brings us to the cancer stem cell hypothesis [90], [91]. While the concept of the cancer stem cell remains controversial [92], [93], here we do not intend to argue for or against this theory. Moreover, we refrain from making specific interpretations of this theory with regards to the exact origins of cancer. It has been argued that there is a distinction between the broader concept of the cancer stem cell on the one hand, and the narrower concept of normal stem cell becoming cancerous [91]. While the cancer stem cell hypothesis states that cancer is maintained by a small fraction of cells with stem-like properties, without making a specific assumption of how those cells are generated, the more narrow theory argues that mutations generated among non-stem cells cannot be cancer-initiating, because (at least, some) cancers originate via the creation of a cancer stem cell, which is a modified stem cell that retains some characteristics of “stemness”.

In the light of this latter hypothesis, let us analyze the process of double-hit mutant production that occurs via mutations in stem cells only. Will our results change if only stem cell mutations can lead to carcinogenic transformation? To accommodate this assumption in our model, we must only use the first term in equation (1). It turns out that in this case, the message remains exactly the same: symmetrically dividing stem cell systems are characterized by a slower production of double-hit mutants compared to asymmetrically dividing stem cells. The universality of this result is explained above: the fate of mutations originating in the differentiated compartment is identical under the two models, and the only difference comes from the fates of mutant stem cells.

### The immortal DNA strand hypothesis

The only scenario where the asymmetrically dividing cells can produce double-hit mutants slower than symmetrically dividing cells is provided by the immortal DNA strand hypothesis [76]. If asymmetrically dividing stem cells are capable of retaining a mutation-free parental copy of the DNA, then the optimal strategy for lowering the change of double-hit mutations is to divide asymmetrically. This theory however remains controversial and further experimental evidence is needed to confirm its applicability *in vivo*, see e.g. [78], [79] for different view points.

### Stochastic tunneling in the context of hierarchical tissue architecture

Our theoretical results on the rate of double-hit mutant formation provide a generalization of a number of previous papers that studied the process of stochastic tunneling. The concept of stochastic tunneling was introduced in Refs. [72], [73] when studying the first step in colon cancer initiation, the inactivation of the tumor suppressor gene APC. The concept has later been investigated by several groups in the context of cancer initiation, escape dynamics [74], and more broadly as a means of crossing an evolutionary valley by an evolving species [75]. The basic Moran process in a homogeneous tissue has been used as the underlying mathematical model. A spatial generalization for the tunneling rate was calculated in Ref. [94], and a generalization to a specific model of renewing epithelial tissue was given in Refs. [88], [95]. The present paper expands the notion of stochastic tunneling to tissues consisting of stem and differentiated cells, whose fate can vary and is governed by relatively complex rules. Formula (1) includes the basic tunneling law of Refs. [72], [73] as a special case, and provides a way to predict the rate of mutant generation based on the stem cell fraction, the mutant fitness, and the probability of symmetric vs asymmetric divisions.

### Oncogenes

This paper considers the inactivation of tumor suppressor genes. An inactivation of the first copy of the tumor suppressor gene does not typically result in the cell breaking out of the homeostatic control. The stem cell-TA cell system continues to function almost normally (except for the one-hit mutants possibly having a smaller or slightly larger fitness compared to the wild-type cells). The overall population remains approximately constant and the cellular turnover continues. A very different picture is observed in the case of oncogene activation. Oncogene activation is normally assumed to confer a significant fitness advantage to the mutated cells, which may lead to a wave of clonal expansion incompatible with homeostasis. In this case, the analysis is quite different because it must describe a population expansion following the oncogene activation.

In order to make the current model useful for describing oncogenes, we can talk about the timing of an oncogene activating mutation under the different assumptions on the symmetry of stem cell divisions. The time distribution of acquiring the first mutant does not depend on whether stem cells divide symmetrically or asymmetrically. The dynamics of the mutant cells however depends on several factors, such as (1) whether wild-type stem cells divide symmetrically or asymmetrically, and (2) how the mutation modifies the growth properties of the cells. If we assume that the cell with an activated oncogene continues to divide in the same fashion as the wild type cell but with a higher frequency, then the following result is observed: (i) under symmetric stem cell divisions, the mutant clone will quickly invade the stem cell population; (ii) under asymmetric divisions, each one-hit mutant will remain in the population, creating a disproportionately large number of mutant TA cells.

### Outlook

The theory developed in this paper is based on a model, which is in some sense an idealization or reality. We assume that stem cells can divide symmetrically and asymmetrically, and calculate which mixture of the two types of divisions minimizes the risk of acquiring double-hit mutants. Under most scenarios, symmetric divisions appear to be the optimal strategy in the context of delaying double-hit mutant generation. Therefore we propose that the reported prevalence of symmetric divisions in certain adult mammalian stem cell systems could have an evolutionary explanation.

We would like to emphasize some of the important simplifications used in the present model. We do not focus specifically on numerous complex aspects of stem cell regulation and functioning. We do not study the mechanisms by which stem cells can switch between asymmetric and symmetric modes of division, and do not extend this model to disease states where the control of stem cell divisions is defective. These are some of important future directions.

In the present paper we considered a two-compartment (stem/TA) model where all non-stem cells were treated as a single type. Our numerical explorations suggest that the addition of more compartments does not change the message of the paper, that is, in the presence of more cell types, symmetric divisions continue to minimize the rate of double-mutant production. Further, the effect of the stem cell niche was modeled in a very basic manner, by assuming the existence of a stem cell compartment and a relatively tight regulation of differentiation vs proliferation decisions. Future directions include the addition of a more detailed description of spatial interactions, and the inclusion of other cellular processes such as de-differentiation.

## Methods

### Numerical simulations

A stochastic numerical simulation was set up according to the following generalized Moran (constant total population) process. The population consists of four types of cells: stem cells (wild-type, 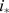, and one-hit mutants, 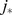), and TA cells (wild-type, 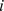, and one-hit mutants, 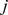). We have 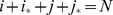, where 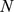 is a constant total population size. The dynamics proceed as a sequence of updates. At each update, one TA cell is randomly removed from the population, and replaced with an offspring of another cell, thus keeping the total population size constant.

The process of division is modeled as follows. All cells (stem or TA cells) have a probability to divide. A cell is chosen for division based on its fitness. The fitness of mutated cells is given by 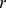 and the fitness of wild-type cells is 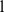. Let us use the notation 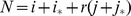. Then the probability that a wild-type stem cell is chosen for division is given by 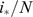; the probability that a mutated stem cell is chosen for division is given by 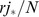; the probability that a wild-type TA cell is chosen for division is given by 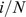; and the probability that a mutant TA cell is chosen for division is given by 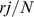.

If a wild-type TA cell divides, it creates another wild-type TA cell with probability 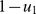, and it creates a one-hit mutant TA cell with probability 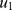. If a mutant TA cell divides, it creates a one-hit mutant TA cell with probability 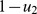, and it creates a two-hit mutant with probability 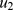. In case of such an event, the process stops.

Divisions of stem cells can be either symmetric (with probability 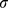) or asymmetric (with probability 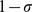). Asymmetric divisions result in a creation of a TA cell. If a wild-type stem cell is dividing asymmetrically, then with probability 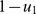 no mutations happen, and a one-hit mutant will be created with probability 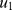. In case of such an event, with probability 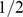 the TA daughter cell will get a mutation, and with probability 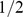 it will be the stem cell that acquires a mutation. Similarly, a one-hit mutant stem cell that divides symmetrically will create a two-hit mutant with probability 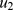, in which case the process stops.

Symmetric divisions can be of two types: a differentiation, which results in a replacement of the dividing stem cell with two TA cells, or a proliferation which results in a creation of a stem cell. The probability of proliferation is taken to be 
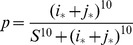
, where 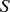 is a constant parameter which measures the expected number of stem cells in the system. The probability of proliferation is given by 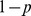. Again, when a wild-type stem cell divides, with probability 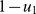 both daughter cells are wild-type, and with probability 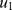 one of the daughter cells is a one-hit mutant. If a one-hit mutant stem cell divides, both daughter cells are one-hit mutants with probability 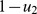, and with probability 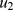 the process stops because a double-hit mutant is created.

**Figure 10:**
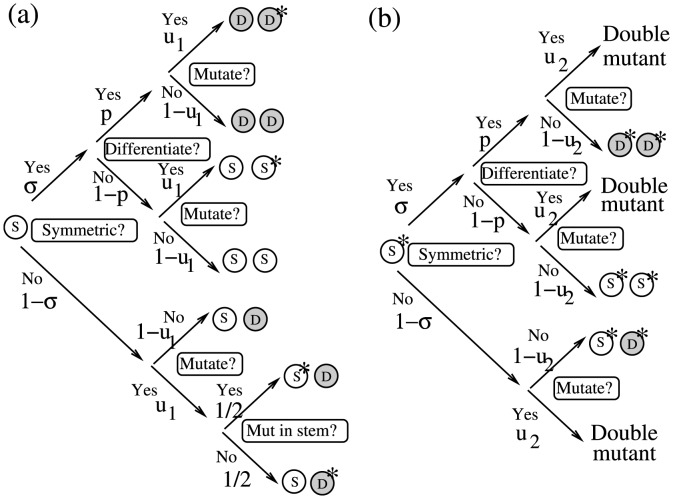
Stem cell division decision trees for the numerical algorithm. (a) Divisions of wild-type stem cells. (b) Divisions of mutant stem cells. Stem cells are denoted by light circles with an “S” and TA cells by shaded circles with a “D”. One-hit mutants are marked with a star.

The decision trees for stem cells are shown in Figure 10, for wild-type stem cells (a) and for mutated stem cells (b). Stem cells are denoted by light circles with “S” and TA cells by shaded circles with “D”. One-hit mutants are marked with a star.

These updates were performed repeatedly until either a double-hit mutant was created, or the maximum number of time-steps was reached, which was set to 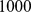. We ran this code for 1000 times. After that we calculated the fraction of runs that resulted in a double-hit mutant, which approximates the probability of double-mutant creation. This quantity was calculated 10 times, and then the averages and standard deviations were calculated.

To simulate the homogeneous Moran process, the same updates were performed except the number of stem cells was zero, 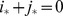.

### Analytical tools

Suppose we have the following version of the Moran process, which consists of a sequence of elementary updates. At each update, a daughter cell is chosen for death at random. Then a cell (a stem cell or a differentiated cell) is chosen to divide, according to its fitness, with mutants having fitness 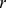. If a differentiated cell is chosen for division, it divides and this concludes the update. If however a stem cell is chosen for division, we proceed as follows. (1) With probability 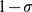, the stem cell can divide asymmetrically, which concludes this step. (2) With probability 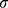, the stem cell divides symmetrically by differentiation, which is followed by a proliferation of another randomly chosen stem cell. Finally, another daughter cell is chosen for death, which concludes this step.

The process described above is slightly different from the numerical agent-based algorithm outlined used in numerical simulations. In the generalized Moran process described here, the numbers of stem cells (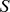) and differentiated cells (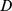) are kept constant at every step. This is a simplification that allowed for analytical tractability (see below). In the numerical simulations the number of stem and differentiated cells fluctuates around a mean value, but despite this difference, the analytical formulas derived here are in an excellent agreement with the simulations.

Note that in order to keep 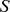 constant, the symmetric stem cell divisions have to come in pairs (one proliferation and one differentiation event), and must be combined with two cell death events. Therefore, on the biological time-scale, an update involving symmetric divisions must have an average duration of two (and not one) elementary updates. Therefore below, when calculating various transition probabilities, the terms associated with symmetric divisions require a factor 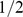.

Let us denote by 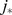 the number of single-mutant stem cells and by 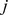 the number of single-mutant differentiated cells. The updates can be envisaged as a Markov process in the space 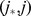, where 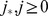, with an additional state 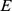 denoting the generation of a double-mutant cell. Below we will use the condition that mutants are drifting at low numbers, 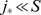 and 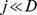. We have the following probabilities:

- The probability that the number of mutant differentiated cells increases by one can be approximated as follows:
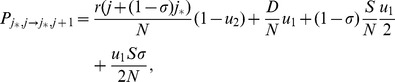
which reflects the following events: (i) a death of a wild-type differentiated cell (probability 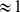), followed by either a faithful division of a mutant differentiated cell, or a faithful asymmetric division of a mutant stem cell; (ii) a division of a wild-type differentiated cell with a mutation; (iii) an asymmetric division of a wild-type stem cell with a mutation happening in the differentiated daughter cell; (iv) a symmetric division of a wild-type stem cell with a mutation (times 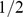 by association with the symmetric division process).
- The probability that the number of mutant differentiated cells decreases by one:
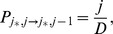
which is the probability that a mutant differentiated cell dies followed by a faithful division of any w.t. cell (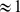).
- The probability that the number of mutant differentiated cells increases by two, and the number of mutant stem cells decreases by one:
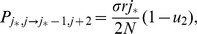
which is only possible for a symmetric update, when two w.t. differentiated cells die (probability 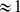) followed by a mutant stem cell differentiating without a further mutation (probability 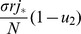), followed by a w.t. stem cell proliferating without a mutation (probability 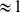); the factor 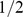 comes from the symmetric update.
- The probability that the number of mutant stem cells increases by one:
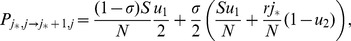
which reflects the following events: (i) following a death of a wild-type differentiated cell (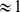), a wild-type stem cell divides asymmetrically with a mutation in the stem cell daughter cell, (ii) a wild-type stem cell proliferates with a mutation (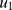), or (iii) a mutant stem cell proliferates without a further mutation (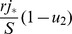).
- The probability to create a double-hit mutant:
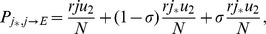
which reflects the following events: (i) a mutant differentiated cell divides with a mutation, (ii) a mutant stem cell divides asymmetrically with a mutation, or (iii) a mutant stem cell undergoes either a differentiation or a proliferation event with a mutation.

Let us define by 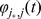 the probability to have 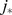 mutated stem cells and 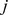 mutated differentiated cells at time 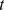. The Kolmogorov forward equation for this function is given by
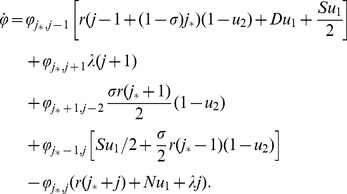
(9)Let us define the probability generating function,
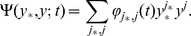
The probability to be in one of the states 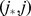 is given by 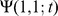. Therefore, the probability to transit to state 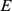 is 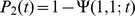. The probability generating function satisfies the following first order PDE, derived by the standard methods (see e.g. [96]):
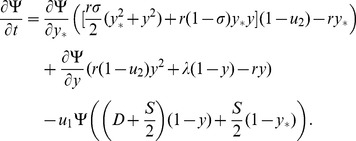
(10)We have
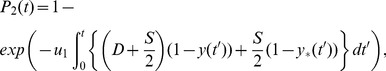
(11)where
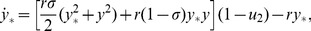
(12)

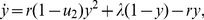
(13)

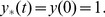
(14)
Equation (11) states that one-hit mutants in differentiated cells are produced by divisions of differentiated cells at the rate 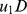 and by divisions of stem cells at the rate 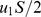. The factor 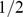 comes from the assumption that in asymmetric divisions, only a half of mutations will be in the differentiated cells (see below for an alternative assumption), and in symmetric divisions which consist of pairs differentiation/proliferation, only half of the time a mutation will happen upon differentiation. Mutations in stem cells are produced by the divisions of stem cells at rate 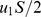. The ordinary differential equations describe the dynamics of lineages that start from one differentiated mutant (equation for 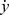) or from one stem cell mutant (equation for 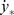). The dynamics of differentiated mutants is independent of 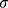.

Let us first solve equation (13), which informs us about the probability of creating a double-hit mutant in a differentiated cell. This Riccati equation can be solved by standard methods, and the growth of the quantity 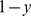 proceeds in the following stages:

- The linear growth stage, where 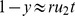, as long as 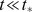 (to be defined).
- The saturation stage, where 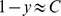, as long as 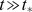.

The constant 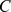 obtained from the stable fixed point of equation (13) is given by the equation
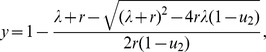
and can be approximated by concise expressions as shown below. Given the solution for 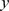, equation (12) can also be analyzed. The function 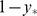 increases monotonically and reaches saturation at 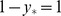, after characteristic time 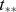. To find that time-scale, we substitute the constant approximation for the function 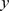, to obtain
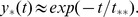
There are several regimes where the expression take a particularly simple form (see Table 1).

#### Regime (2A)

Let us assume that 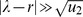, 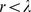, and 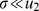. In this case, we have
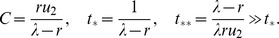
There are therefore three distinct regimes defined by the behavior of the functions 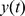 and 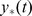.

1. If 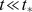, we have 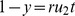 and 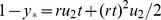. In this case we have
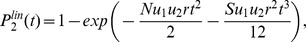
where the second term in the exponent is typically smaller than the first, and the behavior is thus indistinguishable for the usual homogeneous Moran process at early times.
2. If 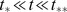, we have 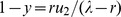 and 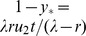. In this case we have
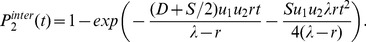
(15)
3. Finally, if 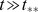, we have 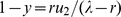 and 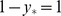, and
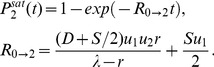
(16)This regime becomes unimportant if for 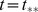 we can show that the quantity in the exponent is much larger than one. We have
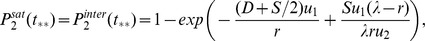
and this quantity is very close to 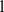 for example if 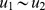 and 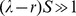.

#### Regime (2B)

Let us assume that 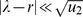 and 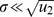. In this case, we have
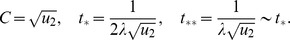
There are therefore only two regimes defined by the behavior of the functions 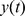 and 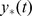.

1. If 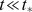, we have as in the previous case, 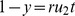 and 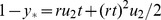. The probability of double-hit mutant production is thus given by
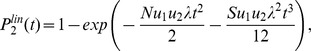
where the second term in the exponent is typically smaller than the first, and the behavior is thus indistinguishable for the usual homogeneous Moran process at early times.
2. If 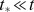, we have 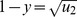 and 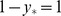. In this case we have
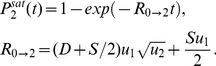
(17)

#### Regime (1A)

Let us assume that 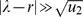, 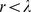, and 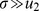. The quantity 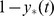 behaves as a linear function,
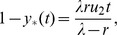
for 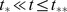, where
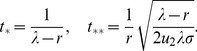
(18)For 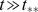, the quantity 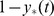 tends to a constant,
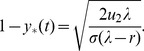
(19)Note that the initial behavior of the function 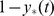 does not depend on 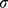. This means that for relatively short times (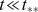), the mutant generation in stem cells proceeds in the same way for symmetric and asymmetric divisions. The length of this regime and the level of saturation however are both functions of 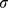. It is easy to see that both 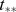 and the saturation level increase as 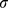 decreases. This means that the rate of mutant accumulation becomes higher for asymmetric divisions.

#### Regime (1B)

Let us assume that 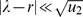 and 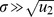. Now, the linear stage for 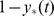 is defined as
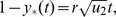
and it occurs for the times 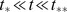, where
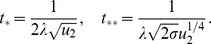
For 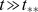, the quantity 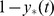 tends to a constant,
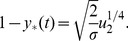
(20)


Calculations for regimes (1C) and (2C) are performed in a similar manner, see Table 2.

#### Alternative mutation mechanisms

In all the previous calculations, we assumed that upon a stem cell division, with probability 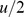 a daughter stem cell acquires a mutation, and with probability 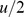 a daughter TA cell acquires a mutation (here, 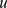 is given by 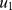 if the dividing cell is wild type, and it is given by 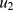 if it is a one-hit mutant). Instead, we now break this symmetry of mutations, and assume that the probability of getting a mutation in a daughter stem cell is 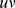 and the probability of getting a mutation in a daughter TA cell is 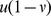, with 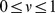. Then, instead of equation (11), we have the following expression:
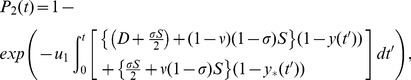
(21)where 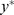 and 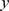 are again given by equations (12) and (13). Equation (21) reduces to equation (11) if 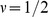. For values of 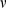 which are above a threshold, 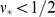, the result remains unchanged such that the probability of double-mutant generation is minimized for 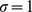 (symmetric stem cell divisions). For smaller values of 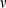, an intermediate value of 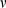 can be optimal, and for very low values of 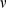, asymmetric divisions provide the lowest probability of double-hit mutant generation.
